# General practitioners’ accounts of negotiating antibiotic prescribing decisions with patients: a qualitative study on what influences antibiotic prescribing in low, medium and high prescribing practices

**DOI:** 10.1186/s12875-019-1065-x

**Published:** 2019-12-10

**Authors:** Marieke M. van der Zande, Melanie Dembinsky, Giovanni Aresi, Tjeerd P. van Staa

**Affiliations:** 1 Centre for Health Informatics, Division of Informatics, Imaging and Data Science, School of Health Sciences, Faculty of Biology, Medicine and Health, University of Manchester, Manchester Academic Health Science Centre, Vaughan House, Portsmouth Road, Manchester, M13 9PL UK; 2 Department of Health Services Research, Institute of Population Health Sciences, University of Liverpool, Liverpool, UK; 3 Health Sciences & Sport, University of Stirling, Stirling, UK; 4 Psychology Department, Università Cattolica del Sacro Cuore, Milan, Italy; 5 Division of Pharmacoepidemiology and Clinical Pharmacology, Utrecht Institute of Pharmaceutical Sciences, Utrecht University, Utrecht, the Netherlands

**Keywords:** Antibiotic prescribing, Attitudes of health care personnel, Primary health care, Shared decision making, Qualitative research

## Abstract

**Background:**

Antimicrobial resistance (AMR) is high on the UK public health policy agenda, and poses challenges to patient safety and the provision of health services. Widespread prescribing of antibiotics is thought to increase AMR, and mostly takes place in primary medical care. However, prescribing rates vary substantially between general practices. The aim of this study was to understand contextual factors related to general practitioners’ (GPs) antibiotic prescribing behaviour in low, high, and around the mean (medium) prescribing primary care practices.

**Methods:**

Qualitative semi-structured interviews were conducted with 41 GPs working in North-West England. Participants were purposively sampled from practices with low, medium, and high antibiotic prescribing rates adjusted for the number and characteristics of patients registered in a practice. The interviews were analysed thematically.

**Results:**

This study found that optimizing antibiotic prescribing creates tensions for GPs, particularly in doctor-patient communication during a consultation. GPs balanced patient expectations and their own decision-making in their communication. When not prescribing antibiotics, GPs reported the need for supportive mechanisms, such as regular practice meetings, within the practice, and in the wider healthcare system (e.g. longer consultation times). In low prescribing practices, GPs reported that increasing dialogue with colleagues, having consistent patterns of prescribing within the practice, supportive practice policies, and enough resources such as consultation time were important supports when not prescribing antibiotics.

**Conclusions:**

Insight into GPs’ negotiations with patient and public health demands, and consistent and supportive practice-level policies can help support prudent antibiotic prescribing among primary care practices.

## Background

Antimicrobial resistance (AMR) is an important public health issue, which poses challenges to patient safety and to the provision of health services [1]. The main driver of AMR is thought to be antibiotic use [2, 3], following antibiotic prescribing by health care professionals. Reducing suboptimal prescribing is crucial for preserving the effectiveness of antibiotics. Approximately 80% of all antibiotic prescribing takes place in primary care [4]. However, clinicians’ prescribing practices have more frequently been studied in inpatient settings [5–9] than in primary care. Studies show that antibiotic prescribing rates vary substantially between primary care practices [10, 11]. This variability cannot be explained by clinical factors alone [11–13]. Although patients’ (e.g., gender, age, ethnicity, and comorbidities) [14–16] and clinicians’ characteristics (e.g., specialty, interest in antibiotic prescribing, professional experience, and emotional state) [5–7, 14, 17–20] influence antibiotic prescribing, no one factor explains it by itself.

The outcomes of consultations in which antibiotics are not prescribed may impact negatively on patients, which plays a role in antibiotic prescribing decisions. Not prescribing is associated with risks of missing a diagnosis, and medico-legal consequences. Particularly in situations where clinical signs are less clear, this may lead to prescribing to be on the safe side [21]. Similarly, prescribing antibiotics may also negatively impact on patients. Risks associated with prescribing include adverse effects of antibiotics, and AMR [22]. However, prescribing is often perceived as less risky than not prescribing [23–30].

Although qualitative studies have addressed antibiotic prescribing in primary care [21, 31–33], there is a lack of in-depth understanding of whether GPs’ perspectives vary with different prescribing levels. Focusing on the three most commonly prescribed infections (upper and lower respiratory tract infections (URTI/LRTI), urinary tract infections (UTI)), the aim of this study is to understand contextual factors related to GPs’ antibiotic prescribing behaviour in low, high, and around the mean (medium) prescribing practices in North-West England.

## Methods

The study was approved by the National Health Services (NHS) England Health Research Authority (IRAS ID 234292), and the University of Manchester Research Ethics Committee (UREC ID 2017–2012-4222).

Semi-structured interviews were conducted with GPs in North-West England. MD, a medical anthropologist (PhD), conducted the first 12 interviews. MZ, a sociologist (PhD) working in health services research with a mostly qualitative research focus, carried out the remaining interviews. With the exception of one interview (which was conducted with two participants simultaneously at the request of the participants), all interviews were one-to-one face-to-face interviews based on NHS premises, mostly in the GP practices the participants are working at. Topics covered during the interview are shown in Table 1. The complete Interview Topic Guide is provided as supplementary documentation (Additional file 1). Participants were instructed to focus primarily on their experiences with URTI/LRTI and UTI, as these are the most common infections consultations are sought for.

**Table 1:** Interview topics

Topics addressed during interview	
• Risk of infection-related complications (such as hospital admission for pneumonia or sepsis)	
• Factors that influence prescribing when facing diagnostic uncertainty	
• Experiences of demand for antibiotic prescribing from patients and carers	
• Perceptions of the relation between antibiotic prescribing and patients’ satisfaction	
• Variability of antibiotic prescribing in general practices	
• Awareness of Anti-Microbial Resistance (AMR)	

The selection of GP practices was based on analyses of publicly available records of prescriptions issued by general practices in the NHS in England for 2016 (“GP Practice Prescribing Presentation-level Data” via NHS Digital, https://digital.nhs.uk/). Using the list size information for each practice, the average sex and age standardised prescribing rate (STAR-PU weightings, http://content.digital.nhs.uk/prescribing/measures) in 2016 was calculated. GP practices with a list size smaller than 750 patients, and practices with standardised prescribing volumes below the 1st centile and above the 99th centile were removed [10]. Further details on practice selection and regional distribution are described in a previous paper [34]. From the remaining dataset of practices (*N* = 466) in North-West England all practices in the bottom 10%, top 10%, and around the mean of the prescribing rates were eligible. Eligible practices were collated in a table by a researcher not involved in the qualitative project. Practices with the same prescribing level were assigned the same number. MD and MZ selected practices to contact from this list. This allowed MD and MZ to be blinded to the practice prescribing status during data collection unless GPs mentioned the practice’s prescribing level during interviews. After the interviews were completed with all participants in a practice, and before analysis started, the interviewers were un-blinded. The aim was to recruit 36 GPs; 12 from low, medium and high prescribing practices respectively as this number was expected to allow us to reach data saturation.

**Fig. 1:**
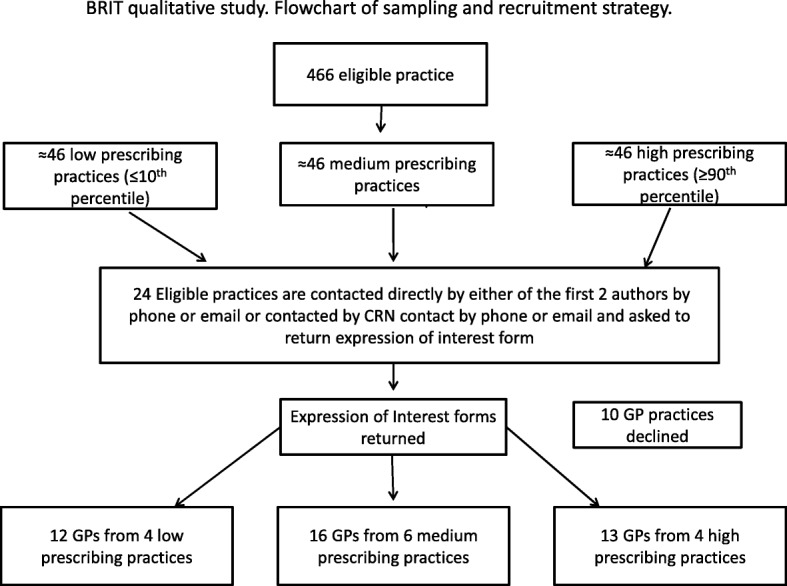
Flowchart of sampling and recruitment strategy

A dual recruitment strategy, including snowballing and local Clinical Research Network-led invitations was used. MD or MZ approached individual GP practices directly or through the project’s Clinical Research Network (CRN) liaison (see Fig. 1 for more detail of the recruitment approach). In their initial contact, MD or MZ or the CRN liaison highlighted that the project was looking to recruit GPs with an aim to maximising diverse representation within each of the three prescribing groups with regards to clinical experience and gender. GPs from individual practices could also make suggestions about who of their colleagues would be interested in participating and share study information materials within their practice. Interested GPs returned an expression of interest form and provided informed written consent prior to the interview. All participants received Amazon vouchers (£70) as remuneration for their time.

All interviews started with questions about the participant’s role in the practice, their clinical experience and the duration of their involvement with the practice. This was meant as an icebreaker and to help contextualise data. Following this, participants were asked about their antibiotic prescribing behaviour with a specific focus on UTI and LRTI/URTI. The order of the questions as shown in the interview topic guide (Additional file 1.1) was not strictly adhered to and participants were allowed to describe their personal experiences of antibiotic prescribing that were relevant to them. Interviews were audio-recorded, transcribed verbatim and thematically analysed.

The initial coding frame was developed from the interview topic guide by MD. MZ did the majority of the coding as the primary investigator. Ten percent of data (four interviews) were coded independently by two researchers (MZ and MD) to ensure coding agreement. The same codes were applied to all transcripts regardless of practice prescribing level (Additional file 1.2). The constant comparative method was used by MZ to develop and refine the codes, compare them across all interview transcripts and compare between the low, medium and high prescribing groups [35]. All codes were subsequently described conceptually and iteratively discussed by the research team to identify cross-cutting themes and highlight differences across prescribing groups. NVivo 11 (QSR International Pty Ltd., 2014. NVivo qualitative data analysis Software) was used to aid in data management, coding and analysis. Discrepancies were resolved through discussion.

No differentiation was made during the coding or analysis between UTI and RTI with regards to GP responses. This was deliberately decided as the focus of the study was on antibiotic prescribing for the most common conditions patients sought consultations for in primary care.

## Results

### Participants

Forty-one GPs from 14 practices representative of low (four practices), medium (six practices) and high (four practices) prescribing practices in a large urban North-West English city were interviewed between January and June 2018. The researchers knew none of the participants prior to the interview. Interviews lasted 20 to 58 min. Participants included GP partners, salaried GPs, registrars and trainees. Trainee doctors have one to 3 years clinical experience, and registrars have an additional one to 2 years clinical experience. Table 2 provides a more detailed overview of participants.

**Table 2:** Characteristics of interview participants

Practice prescribing level	Number of practices	Practice Size	Number of participants	Gender		GP role			
				male	female	Partner	Salaried	Registrar	Trainee
High	4	1 (> 10,000 patients) 2 (8000–10,000 patients) 1 (< 8000 patients)	13	7	6	5	2	0	6
Medium	6	4 (> 10,000 patients) 1 (8000–10,000 patients) 1 (< 8000 patients)	16	5	11	9	2	5	0
Low	4	1 (> 10,000 patients) 2 (8000–10,000 patients) 1 (< 8000 patients)	12	6	6	9	2	0	1
Totals	14		41	18	23	23	6	5	7

We identified three main themes: [1] *Acknowledging patient expectations*, [2] *Reaching decisions in consultations around antibiotic prescribing*, and [3] *Support in prescribing and not prescribing.* The data regarding each of these themes were compared across the low, medium and high prescribing groups. Quotes representative for each theme are included in the text. Each quotation contains an indication of the antibiotic prescribing group and GP’s professional role.

### Acknowledging patient expectations

Participating GPs across all prescribing groups frequently described that they perceived expectations of receiving antibiotics among patients. Though many reported a trend of diminishing demand for antibiotics, about half of the patients seeing the GP for indications where antibiotics may be relevant were perceived to expect antibiotics.

*I think sometimes they want something, I don’t know whether it’s necessarily always antibiotics but it’s a piece of paper that’s to … almost to validate. “I’ve been to the doctors, the doctor thinks I’m ill, now I have a piece of paper, and now I’m walking out and everybody can see that I’m ill.”* (medium prescribing group, participant 5, Registrar)

In fact, GPs sometimes described pressure from patients to prescribe. GPs across the three prescriber groups recounted instances of threatening behaviour from individual patients if antibiotics were withheld.

Participants reported that demand for antibiotics was present among all patient groups. They perceived differences between age groups, with less demand in older than in younger patients, who were perceived as being better at ‘putting up a fight’ and as feeling the need for quickly getting better. Although demand was perceived across all socioeconomic groups, many GPs noticed differences in expectations around antibiotics similar to expectations around other medication. Furthermore, GPs reported that patients’ understanding of AMR varied, and that discussing the importance of AMR was received differently between these groups. Public health campaigns were reported to help in spreading knowledge among all patient groups, but in decision-making this knowledge was not always perceived to be relevant to patients.

### Reaching decisions in consultations around antibiotic prescribing

#### Anticipating patient expectations

Participants reported that GP behaviours in the past often entailed medication prescriptions for various symptoms. This included antibiotic prescriptions for symptoms related to coughs and colds which under current guidelines would not result in antibiotic prescriptions. Many participants reported that this fuelled the expectation among patients of getting a prescription when visiting a doctor and encouraged attendance in the early onset of a disease. Across all prescriber groups, GPs stressed the need for prudent prescribing behaviours to prevent fuelling these expectations. However, GPs in different prescribing groups behaved differently in face of the balance between responding to patient expectations and preventing an increasing spiral of expectations. GPs in the high prescribing group discussed more often that they issued a prescription when perceiving an expectation in patients, whereas GPs in the low prescribing group discussed more often that they stuck with a decision not to prescribe and focused on explaining their decision and acknowledging patients’ suffering. Participants frequently commented that they perceived different behaviours between individual GPs and GP practices.

*And, of course, [when patients are prescribed antibiotics] they get better and it's those doctors that get all the credit. But, in fact, whether they needed antibiotics or not is a question. So it's about putting our curing hat on as doctors to say “no, you don't need them and they'll be more risky and they'll be more harm to you”; or the caring side of us which is “of course you can have whatever you want”.* (low prescribing group, participant 3, partner)

Some GPs reported that empathising with patients who have a virus and acknowledging that they are feeling very unwell may reassure the patient while conveying that antibiotics are not necessary. This was particularly discussed by GPs in the medium and low prescribing groups.

*So something else I’ve been saying to people recently as well is “you can feel just as poorly with a viral infection as you can with a bacterial infection”. And that seems to help people, because they feel like if you don’t send them away with antibiotics they haven’t gone out with a licence to be ill, you know, their doctor said it’s just a virus. So, saying to them, you, you will feel really poorly with this, the only difference is I can’t give you something to make you better.* (medium prescribing group, participant 16, Registrar)

#### Explaining decision-making in consultations

Participants reported that not prescribing antibiotics was more difficult than prescribing, leading to the need for more time in reaching and explaining a decision. Participants from all prescribing groups reported how they discussed their decision-making with patients, while participants from low and medium prescribing practices in particular described how highly detailed and individualised explanations were helpful in getting across decisions not to prescribe antibiotics. In addition to acknowledging patients’ feelings, GPs described how they gave detailed and individualised explanations of clinical findings, as well as making patients aware of their potential to get better by themselves. This included speaking out loud their findings as they proceeded through the examination of a patient, for example temperature and chest sounds, and explaining what the guideline recommendations are for a given combination of findings. In addition, GPs frequently described how they explained that the symptoms should go away in time, while safety-netting for persisting symptoms. GPs also described how in the consultation they informed patients of the risk of side effects of antibiotics and of AMR.

*I say things as they are because I guess even though we’re doctors and we have the power to prescribe or to not prescribe, they have the right to know what kind of bomb they’re having.* (high prescribing group, participant 10, trainee)

*So you educate your patient in terms of: this is why we’re not too certain on giving you antibiotics; you might have a resistance when it’s over, and when you do need it, and it’s more serious, it might not work as well. And a lot of the time they do tend to understand that; it’s just making them aware of what’s going on.* (low prescribing group, participant 4, trainee)

In the high antibiotic prescribing group this was often described as part of ‘scare tactics’, whereas in the low antibiotic prescribing group it was described more in terms of raising patients’ awareness of AMR.

GPs reported that after detailed and individualised explanations patients often, but not always, accept not receiving an antibiotic prescription. Some GPs in the high prescribing group in particular discussed that not prescribing could work against their efforts in reaching a shared decision.

*There’s still patients who will, you know, have made their mind up, they need antibiotics and it is a battle with them.* (medium prescribing group, participant 10, partner)

*But there is that expectation of antibiotics fix all and by saying no, you're belittling their symptoms and not listening. So I see that giving them is an easy way of resolving conflict as well, if that makes sense.* (high prescribing group, participant 6, salaried GP)

Many participants similarly described discussions with patients who insisted on antibiotic prescriptions as a ‘battle’ or a ‘conflict’. When this happened, maintaining the doctor-patient relationship became a central concern, as described in the next section.

#### Maintaining the doctor-patient relationship

Many participants described changing their approach when patients were not convinced after they explained their decision-making. In all antibiotic prescribing groups, antibiotics were sometimes prescribed to maintain the doctor-patient relationship.

*I train junior doctors as well and sometimes, you know, I explain that it’s a case of you might either lose a relationship with a patient, you know, and lose the benefit you could have had in the long term, over an antibiotic prescription. So it’s a difficult balancing act.* (low prescribing group, participant 8, partner)

However, GPs in low and high prescribing practices differed in their description of the circumstances under which they would prescribe antibiotics to patients who insisted on receiving them. Some participants in the low and medium antibiotic prescribing group described giving antibiotics without a clear clinical need, while stating that this should be used sporadically with very demanding patients. GPs in the low and medium antibiotic prescribing group often discussed the need to stick to their clinical decisions, and noticed changes in their style of communicating (as discussed in sections 3.3.1 and 3.3.2), leading to getting their message across more convincingly and thereby reducing the need for such longer discussions. However, this did not always work.

*Despite, if it’s the end of the day of Saturday, I’ll just be completely adamant, and when it’s clear-cut, there’s no budging my rationale. But it has adversely affected our relationship, the doctor/patient relationship.* (medium prescribing group, participant 3, Registrar)

To some GPs in the high prescribing group, sticking with their decision was dependent on high clinical certainty. In the absence of high clinical certainty, and in the interest of maintaining the relationship, prescribing antibiotics was preferred.

*I almost changed my mind halfway through and gave that deferred script [instead of not prescribing]. So … and I think because she was in so much pain I then thought well, is it bacterial? It was very much one sided. So that was a difficult one really. With a bit of pressure from the patient, but a bit of pressure from myself.* (high prescribing group, participant 7, partner)

Here, the participant described issuing a deferred prescription. This involves a prescription given with the advice not to use it unless the patient’s condition deteriorates or fails to improve after a set period. Participants across all prescribing groups reported using deferred prescribing, while many discussed doubt whether deferred prescriptions bring antibiotic use down, as these do not prevent patients from obtaining antibiotics immediately.

In the medium and high antibiotic prescribing group maintaining the doctor-patient relationship was described more often as part of a style of prescribing when a patient clearly voices expectations for antibiotics, and GPs often used a combination of repeating their detailed explanations and prescribing antibiotics. This contrasts with the descriptions of antibiotic prescribing as an exceptional measure among participants in the low prescribing practices.

*So if they've had repeated courses of antibiotics for, say, tonsillitis or something, and they come in wanting more antibiotics, and just expecting to get them because they've always had them, I might start to make noises. About, you know, it's not always the best thing to have antibiotics and this last time, this one time, I'll give you antibiotics but I think you really should think about not taking them. So what that does is, it introduces the concept of not having antibiotics, but it doesn't burn my bridges with them.* (medium prescribing group, participant 4, partner)

### Support in prescribing and not prescribing

GPs across all prescriber groups described that not giving antibiotics required more confidence and experience of the GP, more resources within the practice and more support from the wider health system. Many participants perceived, moreover, that confidence was linked to feeling trusted and backed up by others in their decisions.

*I think there needs to be a bit more trust in the medical, clinical decision-making that is done by GPs, which would then, I think, promote their confidence a lot more, as well, in saying no to patients.* (low prescribing group, participant 4, trainee)

Having resources available for bringing patients back in made not prescribing safer for the GP, whereas not having time or being at the end of a very busy day increased prescribing.

*I think time is … time is a killer of … of those consultations and if I have six extras they're far more likely to get antibiotics than if I have no extras.* (high prescribing group, participant 2, partner)

Especially in the winter months when the demand for antibiotics rises, GPs across prescribing groups described fatigue and moments of not feeling able to face another intense discussion. In such periods, bringing patients back in was often not possible as the demand for appointments was high. In addition, prescribing was then seen as a way to maintain patient safety, particularly in the absence of resources to do so by other means.

*It sounds awful but when you’re running late or you have very tight timings and it’s almost a) safer and b) sort of better for the patient and easier to just give them something and get them out.* (high prescribing group, participant 13, trainee)

Some GPs in one of the practices in the low prescribing group described that in their practice the decision was made to extend the time of the consultations. This was reported to be helpful in explaining decisions and reaching agreement in conversations with patients. In addition, some practices had a triage system, taking some of the pressure off the number of consultations per day further enabled GPs to prescribe less, while enabling time to bring patients in to the practice if necessary.

GPs who worked in low prescribing practices often discussed the need for and use of measures to support GPs in not prescribing antibiotics.

*And so, you know, if there’s … if there’s something for me, out of all of this, is that, if we don’t get the demand management right as a system, we can do what we want with the practitioner, we’re not going to solve the problem. This is absolutely about … allow the headspace for, for decision fatigue not to take place.* (low prescribing group, participant 7, partner)

GPs saw a role for public health messages to allow this headspace, but also discussed practice-level support. Particularly tools and resources that show the need or absence of need for antibiotics were reported as useful, both as a visual aid and as a means of validating the GPs’ decision by an outside, independent source.

*Yeah, and then it backs up your decision a little bit more. [ … ] So, if [a tool could show] if a 30 year old comes with a chest infection, and their observations are normal, most of them will clear the infection without needing antibiotics, then that would massively change my practice, ‘cause I’d be much more confident.* (medium prescribing group, participant 15, Registrar)

In addition, insight into their own prescribing levels was noted as a useful resource by some GPs. Participants pointed out that prescribing levels were influenced by many factors, and insight into their own prescribing would be useful, but only if it was sensitive enough to the context of prescribing, for example the practice setting and types of indications patients attended for. In one of the low prescribing practices, GPs were made aware of their prescribing of broad-spectrum antibiotics by obliging GPs to give a reason for every broad-spectrum antibiotic prescribed. Being able to consult regularly with a microbiologist or a pharmacist affiliated to the practice further supported GPs in their prescribing decision-making and assessment in complicated cases, and was particularly discussed by GPs in low and medium prescribing practices.

In the low and medium prescribing practices, GPs often reported that they did not feel alone in their decision-making, being supported within the practice by their colleagues. GPs then knew that their colleagues had similar antibiotic prescribing behaviour, and similar discussions with patients.

*I think because we all prescribe fairly similarly it’s unlikely that they’re going to get something different from somebody else a day or two days later. And I think...I think patients are learning over time.* (medium prescribing group, participant 9, partner)

In the high prescribing practices, GPs described doing their decision-making on their own. Many GPs in this group were either not sure if the other GPs in the practice would make similar decisions or noted that patients might get antibiotics from another GP in the practice.

*I think my initial training practice, we didn't use to use as many antibiotics. And I think there was that culture of not, and I think we're in a culture of using. [ … ]I think if there were lots of doctors not prescribing, it'd be easier. So it's trying to change that culture really. And, and also I think in the past maybe doctors have prescribed. So the patient will say well, “I always come in and they give me this and”, and so it's changing that as well.* (high prescribing group, participant 7, partner)

If colleagues in the practice regularly prescribed patients antibiotics even when not clinically needed, GPs reported that patients’ expectations had been raised to the point where they were not able to explain a decision not to prescribe to a patient.

Many GPs described reducing their prescribing over time, as they grew more confident and experienced in clinical practice and built up a rapport with their patients. Some GPs did not note a change in their prescribing behaviours, and one GP described increased prescribing after experiences with a (non-antibiotics-related) adverse event and a subsequent complaint. Adverse events and complaints had a deeply felt impact on GPs and could lead to changing prescribing behaviour after the event. Some GPs in the high antibiotic prescribing group described thinking that if a patient was adamant that they wanted antibiotics, they had to prescribe to avoid complaints or doubt about their decision in case of adverse events. In the low and medium antibiotic prescribing group, complaints or adverse events were also described as having deeply felt effects, but after these experiences GPs tended towards continuing take ample time for giving explanations, and towards deferred prescribing. GPs across all prescribing groups noted a lack of support for their decision-making by the wider health care system, and not feeling backed up in case of complaints or adverse events.

*But I'm gonna play defensive and give a prescription to avoid a complaint, because complaints are so time consuming, stressful, and, at the end of the day, one is … I'm in doubt that I'm gonna get support.* (low prescribing group, participant 2, partner)

Participants from high prescribing practices tended to report fewer resources to support GPs in their prescribing decisions. In practices where for example more locums were employed or time for regular meetings was less available, addressing prescribing variations was reported as more difficult. GPs discussed that practices attempting to change their prescribing rates often focused on auditing and monitoring the prescribing rates in the practice. In some practices, monitoring was regularly performed, and meetings often addressed discussions of antibiotic prescribing. GPs found this helpful not only in getting information, but also in reflecting on their own prescribing.

*So I know we have our weekly meeting here, where anything that – even if it’s just a small thing – it will be brought up informally. I think having that constant or regular communication, I think, will help things massively.* (medium prescribing practice, participant 3, registrar)

Thus, GPs reported a mix of monitoring and having resources such as consultation time, case discussions, and support both within the practice and in the wider health care system as important ingredients in optimizing antibiotic prescribing.

## Discussion

Our data confirm that drivers of antibiotic prescribing are interrelated and not one factor alone can describe it. GPs in practices with varying levels of prescribing described that experience and confidence in clinical decision-making are as important as acknowledging a patient’s concerns and arriving at a shared decision during a consultation that is both clinically appropriate and satisfying to the patient. In addition, GPs’ accounts suggest that antibiotic prescribing is an area of potential tension in the relationship between GP and patient and particularly in the communication after a clinical decision has been made. GPs in higher and lower prescribing practices perceived such potential tensions, but differed in ways of addressing these in their decision-making, and in the wider support and resources available to them. In high prescribing practices, GPs noted difficulties in not prescribing antibiotics due to variations in prescribing within the practice, and a consequent increase in, or reinforcement of, patient expectations to receive an antibiotic prescription. In low prescribing practices, GPs noted the importance of sufficient support or resources to enable them to make a strong case for prescribing only when clinically needed and managing possible tension with patients’ expectations. GPs in high prescribing practices described not having enough support and mentioned fewer resources available to them than GPs in low prescribing practices. Reducing prescribing rates is difficult for individual GPs to achieve without support within the practice and wider healthcare system.

Patients’ or their representatives’ expectations regarding receiving antibiotics have an equally important role in shaping shared decision-making. GPs often perceive patients’ [23, 26, 36, 37], or parents’ and carers’ (when the patient is a child) [22, 23, 27, 38, 39] expectations to be prescribed an antibiotic, and may overestimate these [24]. Expectations may involve an explicit or implicit request for an antibiotic [25, 39–41], though not all patients have such requests [23, 40, 42]. Participants in this current study stated that they saw a decline in expectations for prescription of antibiotics but perceived considerable expectations to remain. Particularly, GPs described that messages about AMR were not received or understood equally across different population groups (based on age and socioeconomic status). Public health messages were felt to be helpful here, but sometimes limited in effectiveness. Public health campaigns about AMR potentially reduce requests for antibiotics, but may also have the opposite effect of increasing requests among some patients [43]. In addition, GPs in high prescribing practices discussed that discrepancies in prescribing antibiotics among GPs in a practice could maintain patients’ expectations high.

Antibiotic prescribing plays a significant role in the context of maintaining and strengthening the doctor-patient relationship. GPs express a perceived need to offer something tangible such as a pill [24–27, 36, 42, 44, 45] or a prescription to meet patients’ expectations. However, offering a pill without offering reassurance, desired information, or addressing the symptoms patients were concerned about does not seem to increase satisfaction [25]. These sentiments were reiterated in our data where GPs expressed the need to acknowledge the illness both through verbal affirmation of symptoms the patient described, but also in a more tangible way. This was highlighted in discussions of the usefulness of outside resources or tools which would help visualise to the patient the diagnostic process as substitutional elements for a prescription. Deferred prescriptions could also help here, as discussed by some GPs. Participants in our study expressed doubting whether deferred prescribing brings antibiotics use down, as patients could still use the deferred script to get antibiotics immediately, but also acknowledged that it could lower prescribing compared to an immediate antibiotics prescription. Indeed, other studies have found that deferred prescriptions often lead to antibiotics use [46]. Although deferred antibiotic prescriptions may reduce use compared to immediate prescriptions, not prescribing is more effective in reducing use and thus, other strategies such as the tools described above and communication strategies may be more effective [43, 46, 47].

Besides outside resources or tools to help visualise the diagnostic process, acknowledging the patient’s concerns and symptoms through reassurance and highlighting that a virus can make one feel very ill were seen as effective strategies by participants from low and medium prescribing practices. Reaching decisions in consultations where antibiotic prescribing is an option and styles of communication in doing so was of central concern to the GPs in this study. Patient-centeredness in this communication is increasingly considered important [48], particularly through shared decision-making [48, 49]. Communication-based interventions aimed at the general public have been shown to be effective in reducing antibiotic prescribing, both through population-level interventions and through clinician-led interventions [50]. However, more work is needed to identify the most effective communication strategies, and determine their ‘active ingredients’ which bring about reduced antibiotic use [51]. In our study, GPs often tried varying degrees of openness and styles of communicating in discussing decision-making with patients. In reaching a shared decision, time for giving detailed explanations and translating the population-level message of AMR reduction to an individual level were important strategies.

Besides the factors described above, factors related to the organization and management of primary care practices, such as time pressure [23–26, 52, 53], and particularly the limited time available for a consultation with a patient [24–27, 54] are linked to increased antibiotic prescribing in primary care. In addition, encouragement of intra-professional discussion from a management level, internalized guidelines, and common management of patient expectations across the practice may enable GPs to prescribe less [33]. Our data indicates these elements to be present in low prescribing practices. Participants from low prescribing practices reported that these were crucial resources and support mechanisms which enabled them to reduce their prescribing rates and maintain these lower levels, feeling confident that their decisions will be supported by colleagues within the practice.

One possible solution to monitoring antibiotic prescribing is the development of software for this purpose. Lee, John and Lovinsky [55] have shown the effectiveness of such a tool for antimicrobial stewardship in an acute care community hospital setting. Future research should examine the effectiveness of such a tool within a primary care setting.

### Strengths and limitations

To our knowledge, this is the first study exploring GP perspectives about antibiotic prescribing and AMR awareness with a purposive sample of high, medium and low prescribers. The prescribing group was determined based on practice prescribing levels adjusted for patient characteristics, based on openly available prescribing data. It was not possible to determine individual GP prescribing levels and prescribing behaviours may differ between GPs within a practice. We tried to address this by interviewing several GPs from the same practice. In addition, our findings suggest that practice-level support and resources have a large role in GPs’ prescribing. An individual-level analysis may not have shed light on this.

The study was conducted in a former industrial city in North-West England, which is densely populated with a vibrant ethnically diverse population. It remains one of the biggest economic centres in the UK. This local context may vary from other regions and may have influenced the results. In particular, the prevalence of comorbidities as well as the presence of patient expectations for antibiotics may be higher in this region than in some other areas. In addition, in other regions, differences in contextual factors (such as free prescriptions in Scotland) may influence prescribing. However, earlier studies have found similar influences across other regions, and the organisational factors identified are likely to be similar in other regions. We acknowledge that the questions asked are very specific and could be interpreted as leading. We further acknowledge that the sole focus on GPs as prescribers is a limitation as there might have been other prescribers who could have a substantial impact on prescribing levels for acute illness. The influences on prescribing found in this study point to aspects of primary care where antibiotic prescribing can be improved, especially in areas with high need or high variability.

## Conclusions

This study shows that influences on antibiotic prescribing are interrelated, and centre on communication between doctor and patient, and addressing patients’ expectations in decision-making. Increasing dialogue in the practice, increasing consistency of prescribing between GPs within the practice, supportive practice policies, and enough resources such as consultation time and outside support such as visual tools presenting optimal prescribing decisions were important supports when not prescribing antibiotics. Financial incentives have been provided on a national level since 2015 as part of the Quality Premium NHS initiative. Recent research has shown that these incentives appear to reduce age-related antibiotic prescribing with seasonal variations [56]. We agree with these authors that prescribing rates should be monitored to ensure incentives are not negatively influencing decision-making in cases of clinical uncertainty, leading to under-prescribing, particularly for lower RTIs. Our findings suggest that incentives aimed at increasing support, increasing dialogue within the practice to enable reductions in variation, and enabling confidence in decision-making regarding antibiotic prescribing might be more promising avenues for changing prescribing than incentives aimed at prescribing level outcomes. Our findings suggest that monitoring prescribing within a practice may support reductions in prescribing within a practice, when paired with supportive policies and enhancing intra-professional discussions within a practice.

## Supplementary information

**Additional file 1.** Supplementary information to“General practitioners’ accounts of negotiating antibiotic prescribing decisions with patients: a qualitative study on what influences antibiotic prescribing in low, medium and high prescribing practices”.

## Data Availability

The data generated for this study are not made publicly available, due to protection of the anonymity of study participants. All participants in the study consented to the use of pseudonymised quotations, but consent was not obtained for the public availability of full interviews.

## Abbreviations

AMR: Antimicrobial resistance
GP: General practitioner
LRTI: Lower respiratory tract infection
NHS: National Health Service
UK: United Kingdom
URTI: Upper respiratory tract infection
UTI: Urinary tract infection

## References

[1] World Health Organization (2015). Global Action Plan on Antimicrobial Resistance.

[2] Costelloe C, Metcalfe C, Lovering A, Mant D, Hay AD (2010). Effect of antibiotic prescribing in primary care on antimicrobial resistance in individual patients: systematic review and meta-analysis. BMJ.

[3] Goossens H, Ferech M, Vander Stichele R, Elseviers M (2005). Outpatient antibiotic use in Europe and association with resistance: a cross-national database study. Lancet.

[4] Standing Medical Advisory Committee (Sub-Group on Antimicrobial Resistance) (1998). The path of least resistance.

[5] Menendez R, Torres A, Zalacain R, Aspa J, Martin-Villasclaras JJ, Borderias L (2005). Guidelines for the treatment of community-acquired pneumonia: predictors of adherence and outcome. Am J Respir Crit Care Med.

[6] Halm EA, Switzer GE, Mittman BS, Walsh MB, Chang C-CH, Fine MJ (2001). What factors influence Physicians' decisions to switch from intravenous to Oral antibiotics for community-acquired pneumonia?. J Gen Intern Med.

[7] Avorn J, Solomon DH (2000). Cultural and economic factors that (mis) shape antibiotic use: the nonpharmacologic basis of therapeutics. Ann Intern Med.

[8] Pouwels KB, Hopkins S, Llewelyn MJ, Walker AS, McNulty CA, Robotham JV (2019). Duration of antibiotic treatment for common infections in English primary care: cross sectional analysis and comparison with guidelines. BMJ.

[9] Gharbi M, Drysdale JH, Lishman H, Goudie R, Molokhia M, Johnson AP (2019). Antibiotic management of urinary tract infection in elderly patients in primary care and its association with bloodstream infections and all cause mortality: population based cohort study. BMJ.

[10] Wang KY, Seed P, Schofield P, Ibrahim S, Ashworth M (2009). Which practices are high antibiotic prescribers? A cross-sectional analysis. Br J Gen Pract.

[11] Li Yan, Mölter Anna, White Andrew, Welfare William, Palin Victoria, Belmonte Miguel, Ashcroft Darren M, Sperrin Matthew, van Staa Tjeerd Pieter (2018). Relationship between prescribing of antibiotics and other medicines in primary care: a cross-sectional study. British Journal of General Practice.

[12] Fischer T, Fischer S, Kochen MM, Hummers-Pradier E (2005). Influence of patient symptoms and physical findings on general practitioners’ treatment of respiratory tract infections: a direct observation study. BMC Fam Pract.

[13] Hajjaj FM, Salek MS, Basra MKA, Finlay AY (2010). Nonclinical influences, beyond diagnosis and severity, on clinical decision making in dermatology: understanding the gap between guidelines and practice. Br J Dermatol.

[14] Barlam TF, Morgan JR, Wetzler LM, Christiansen CL, Drainoni ML (2015). Antibiotics for respiratory tract infections: a comparison of prescribing in an outpatient setting. Infect Control Hosp Epidemiol.

[15] Xu KT, Roberts D, Sulapas I, Martinez O, Berk J, Baldwin J (2013). Over-prescribing of antibiotics and imaging in the management of uncomplicated URIs in emergency departments. BMC Emerg Med.

[16] Gerber JS, Prasad PA, Localio AR, Fiks AG, Grundmeier RW, Bell LM (2013). Racial differences in antibiotic prescribing by primary care pediatricians. Pediatrics.

[17] Bharathiraja R, Sridharan S, Chelliah LR, Suresh S, Senguttuvan M (2005). Factors affecting antibiotic prescribing pattern in pediatric practice. Indian J Pediatr.

[18] Livorsi D, Comer A, Matthias MS, Perencevich EN, Bair MJ (2015). Factors influencing antibiotic-prescribing decisions among inpatient physicians: a qualitative investigation. Infect Control Hosp Epidemiol.

[19] Nyquist AC, Gonzales R, Steiner JF, Sande MA (1998). Antibiotic prescribing for children with colds, upper respiratory tract infections, and bronchitis. JAMA.

[20] Parker HM, Mattick K (2016). The determinants of antimicrobial prescribing among hospital doctors in England: a framework to inform tailored stewardship interventions. Br J Clin Pharmacol.

[21] Germeni E, Frost J, Garside R, Rogers M, Valderas JM, Britten N (2018). Antibiotic prescribing for acute respiratory tract infections in primary care: an updated and expanded meta-ethnography. Br J Gen Pract.

[22] Elwyn G, Gwyn R, Edwards A, Grol R (1999). Is 'shared decision-making' feasible in consultations for upper respiratory tract infections? Assessing the influence of antibiotic expectations using discourse analysis. Health Expect.

[23] Barden LS, Dowell SF, Schwartz B, Lackey C (1998). Current attitudes regarding use of antimicrobial agents: results from physician's and parents' focus group discussions. Clin Pediatr (Phila).

[24] Kumar S, Little P, Britten N (2003). Why do general practitioners prescribe antibiotics for sore throat? Grounded theory interview study. BMJ.

[25] Butler CC, Rollnick S, Pill R, Maggs-Rapport F, Stott N (1998). Understanding the culture of prescribing: qualitative study of general practitioners’ and patients’ perceptions of antibiotics for sore throats. BMJ.

[26] Björnsdóttir I, Hansen EH (2002). Intentions, strategies and uncertainty inherent in antibiotic prescribing. Eur J Gen Pract.

[27] Petursson P (2005). GPs’ reasons for “non-pharmacological” prescribing of antibiotics. A phenomenological study. Scand J Prim Health Care.

[28] Klein EY, Martinez EM, May L, Saheed M, Reyna V, Broniatowski DA (2017). Categorical risk perception drives variability in antibiotic prescribing in the emergency department: a mixed methods observational study. J Gen Intern Med.

[29] Duane S, Domegan C, Callan A, Galvin S, Cormican M, Bennett K (2016). Using qualitative insights to change practice: exploring the culture of antibiotic prescribing and consumption for urinary tract infections. BMJ Open.

[30] Vazquez-Lago JM, Lopez-Vazquez P, Lopez-Duran A, Taracido-Trunk M, Figueiras A (2012). Attitudes of primary care physicians to the prescribing of antibiotics and antimicrobial resistance: a qualitative study from Spain. Fam Pract.

[31] Tonkin-Crine S, Yardley L, Little P (2011). Antibiotic prescribing for acute respiratory tract infections in primary care: a systematic review and meta-ethnography. J Antimicrob Chemother.

[32] Teixeira Rodrigues A, Roque F, Falcao A, Figueiras A, Herdeiro MT (2013). Understanding physician antibiotic prescribing behaviour: a systematic review of qualitative studies. Int J Antimicrob Agents.

[33] Strandberg EL, Brorsson A, Andre M, Grondal H, Molstad S, Hedin K (2016). Interacting factors associated with low antibiotic prescribing for respiratory tract infections in primary health care - a mixed methods study in Sweden. BMC Fam Pract.

[34] Molter A, Belmonte M, Palin V, Mistry C, Sperrin M, White A (2018). Antibiotic prescribing patterns in general medical practices in England: does area matter?. Health Place.

[35] Glaser BG, Strauss AL, Glaser BG, Strauss AL (1967). The constant comparative method of qualitative analysis. The discovery of grounded theory: strategies for qualitative research.

[36] Rose PW, Ziebland S, Harnden A, Mayon-White R, Mant D (2006). Why do general practitioners prescribe antibiotics for acute infective conjunctivitis in children? Qualitative interviews with GPs and a questionnaire survey of parents and teachers. Fam Pract.

[37] Altiner A, Knauf A, Moebes J, Sielk M, Wilm S (2004). Acute cough: a qualitative analysis of how GPs manage the consultation when patients explicitly or implicitly expect antibiotic prescriptions. Fam Pract.

[38] Tonkin-Crine S, Yardley L, Coenen S, Fernandez-Vandellos P, Krawczyk J, Touboul P (2011). GPs’ views in five European countries of interventions to promote prudent antibiotic use. Br J Gen Pract.

[39] Everitt H, Kumar S, Little P (2003). A qualitative study of patients' perceptions of acute infective conjunctivitis. Br J Gen Pract.

[40] Hawkings NJ, Butler CC, Wood F (2008). Antibiotics in the community: a typology of user behaviours. Patient Educ Couns.

[41] Larson EL, Dilone J, Garcia M, Smolowitz J (2006). Factors which influence Latino community members to self-prescribe antibiotics. Nurs Res.

[42] Jonsson H, Haraldsson RH (2002). Parents' perspectives on otitis media and antibiotics. A qualitative study. Scand J Prim Health Care.

[43] Edwards M, Dennison J, Sedgwick P (2003). Patients’ responses to delayed antibiotic prescription for acute upper respiratory tract infections. Br J Gen Pract.

[44] Kenealy T, Arroll B (2013). Antibiotics for the common cold and acute purulent rhinitis. Cochrane Database Syst Rev.

[45] Hart AM, Pepper GA, Gonzales R (2006). Balancing acts: deciding for or against antibiotics in acute respiratory infections. J Fam Pract.

[46] Francis NA, Gillespie D, Nuttall J, Hood K, Little P, Verheij T (2012). Delayed antibiotic prescribing and associated antibiotic consumption in adults with acute cough. Br J Gen Pract.

[47] Peters S, Rowbotham S, Chisholm A, Wearden A, Moschogianis S, Cordingley L (2011). Managing self-limiting respiratory tract infections: a qualitative study of the usefulness of the delayed prescribing strategy. Br J Gen Pract.

[48] Singh Ospina Naykky, Phillips Kari A., Rodriguez-Gutierrez Rene, Castaneda-Guarderas Ana, Gionfriddo Michael R., Branda Megan E., Montori Victor M. (2018). Eliciting the Patient’s Agenda- Secondary Analysis of Recorded Clinical Encounters. Journal of General Internal Medicine.

[49] Legare F, Adekpedjou R, Stacey D, Turcotte S, Kryworuchko J, Graham ID (2018). Interventions for increasing the use of shared decision making by healthcare professionals. Cochrane Database Syst Rev.

[50] Cross EL, Tolfree R, Kipping R (2017). Systematic review of public-targeted communication interventions to improve antibiotic use. J Antimicrob Chemother.

[51] McParland JL, Williams L, Gozdzielewska L, Young M, Smith F, MacDonald J (2018). What are the ‘active ingredients’ of interventions targeting the public’s engagement with antimicrobial resistance and how might they work?. Br J Health Psychol.

[52] Coenen S, Van Royen P, Vermeire E, Hermann I, Denekens J (2000). Antibiotics for coughing in general practice: a qualitative decision analysis. Fam Pract.

[53] Bjorkman I, Berg J, Roing M, Erntell M, Lundborg CS (2010). Perceptions among Swedish hospital physicians on prescribing of antibiotics and antibiotic resistance. Qual Saf Health Care.

[54] Palmer DA, Bauchner H (1997). Parents’ and physicians’ views on antibiotics. Pediatrics.

[55] Lee A, John S, Lovinsky R (2018). Surveillance software and prospective audit and feedback rounds advance antimicrobial stewardship at an acute care community hospital. Healthc Q.

[56] Bou-Antoun S, Costelloe C, Honeyford K, Mazidi M, Hayhoe BWJ, Holmes A (2018). Age-related decline in antibiotic prescribing for uncomplicated respiratory tract infections in primary care in England following the introduction of a national financial incentive (the quality premium) for health commissioners to reduce use of antibiotics in the community: an interrupted time series analysis. J Antimicrob Chemother.

