# Supplementary material for: General practitioners’ accounts of negotiating antibiotic prescribing decisions with patients: a qualitative study on what influences antibiotic prescribing in low, medium and high prescribing practices
Source: BMC Fam Pract. 2019 Dec 10;20:172. doi: 10.1186/s12875-019-1065-x (PMC6905031; doi:10.1186/s12875-019-1065-x)
Supplement: Supplementary file 1 — Additional file 1. Supplementary information to“General practitioners’ accounts of negotiating antibiotic prescribing decisions with patients: a qualitative study on what influences antibiotic prescribing in low, medium and high prescribing practices”. [file 12875_2019_1065_MOESM1_ESM.pdf]

Additional file 1.

Supplementary information to

"General practitioners' accounts of negotiating antibiotic prescribing decisions with patients: A qualitative study on what influences antibiotic prescribing in low, medium and high prescribing practices"

Additional file 1.1

Topic guide

1. Pre-amble to the interview

2. Introductory questions

2.1 Could you briefly introduce yourself (do not say your full name)? Some general information on what is your professional role and what is your experience as a GP would be sufficient.

> ***Probes**. What's your role within the practice (GP partner, salary GP, GP trainee)? How many years of experience do you have? Do you have experience in training other GPs?*

2.2 The interviewer introduces the following section of the interview and explains that questions will be limited for parsimony to the three most prescribed diseases: upper respiratory tract infection (URTI), lower respiratory tract infection (LRTI), and urinary tract infection (UTI).

3. Risk of infection-related complications (such as hospital admission for pneumonia or sepsis)

3.1 How important is a patient's risk of complications such as hospital admission in your decision to prescribe an antibiotic?

3.2 Recent research has found that there is a substantive variability in the risk of infection-related complications but that the decision to prescribe antibiotics was not related to this. Patients with very low risks (such as younger otherwise healthy adults) often got antibiotics while patients with high risks (such as elderly patients with co-morbidity) were sometimes left untreated. What do you think could be the reasons that explain this?

4. Factors that influence prescribing when facing diagnostic uncertainty

4.1 Could you briefly describe a recent relevant case of URTI, LRTI or UTI in which you were uncertain on whether or not it was appropriate to prescribe an antibiotic?

> ***Probes**. How was the patient? Can you describe what motivated your decision in that particular occasion? Was it how the patient described their symptoms or was how worried the patient was? What was the outcome? Was an antibiotic prescribed?*

4.2 Did you feel you had enough time to do the consultation?

> ***Probes**. Did you feel rushed? How long did the consultation last?*

4.3 What guidelines on antibiotic prescribing (AP) most influence your work? (e.g., national guidelines on AP, local clinical commissioning group CCG policy, or others).

> ***Probes**. Can you tell me more about the reasons why you consider one more influential than the others?*

4.4 Do you ever consult other GPs' for opinions and past experiences of AP decision making?

4.5 What could help you in optimising antibiotic prescribing for common infections?

***Probes**. Point-of-care testing? Simple home monitoring of patients? Prediction of risk of hospital admission based on a patient's characteristics?*

5. Experiences of demand to AP from patients and carers

5.1 Would you say that URTI, LRTI or UTI patients expect to be prescribed an antibiotic?

> ***Probes**. Frequent or rarely? Can you describe a recent occasion in which this has happened? How the patient/carer expressed their wishes? What reasons did they give? How do you think that affected your decision?*

6. Perceptions of the relation between AP and patients' satisfaction;

6.1 What would you say are patients' main expectations around a consultation for URTI, LRTI or UTI?

6.2 Would you consider that the decision to prescribe an antibiotic has an impact on patients' satisfaction around the consultation?

7. Variability of antibiotic prescribing in general practices

7.1 Recent research has found that there is substantive variability in the level of antibiotic prescribing for common infections such as URTI, LRTI or UTI. In some practices, only 20% of patients get an antibiotic while in others more than 80% do. What do you think could be the reasons that explain this?

7.2 Recent research in UK general practices has found that the practices that prescribe lots of antibiotics see their patients more frequently (higher consultation rates) and have shorter durations of consultations. Do you think that practice characteristics are important drivers for antibiotic prescribing and what are these?

7.3 The same research also found that the practices that prescribe lots of antibiotics are prescribed more others drugs, including pain killer and benzodiazepines. What do you think is happening?

7.4. Recent research has found that there is a substantive regional variability in antibiotic prescribing. Practices in Greater Manchester prescribe more antibiotics than practices in London. What explains this?

8. Awareness on Anti-Microbial Resistance (AMR)

8.1 Are you aware of any particular AMR in the local area?

> ***Probes**. Have you experienced any forms of resistance to antibiotics in your patient population?*

8.2 How does that knowledge affect your decision on whether you should prescribe an antibiotic? And which antibiotic should be prescribed?

8.3 How do you address AMR within your practice?

8.4. What do you think GPs can do to reduce AMR at the local level?

Additional file 1.2

Codebook

Name
acting on AMR
auditing as means of monitoring AP
awareness of AMR among GPs
changes in local guidelines due to AMR
disease groups with resistance
factors impacting on creating AMR
GPs role in addressing AMR
choosing different types of AB
role of GPs in patient education around AMR
impact of AMR awareness on prescribing
choosing type of AB based on resistance likelihood
finding AP reduction reasonable
role of public health in raising patient awareness of AMR
communicating with patients
communicating with patients as a battle
doctor-patient relationship
giving AB if patient cannot be dissuaded
giving detailed explanations
link fulfilled patient expectations and satisfaction
managing patient expectations with foresight
prescribing to end the discussion

Name
taking the time for communicating decision-making
using AB complications as bargaining tool
decision-making processes
assessing patient's self-care abilities
being aware of risk profile when prescribing
case-by-case decision-making
combining evidence sources to reach decision
dealing with clinical uncertainty
always making up a decision
consultations when decision is easy
deferred prescribing
frequently being uncertain
prescribing happens defensively
safety netting to address uncertainty
strategies when risks are uncertain
uncertainty interpreting lab results
deciding based on clinical experience
diverging from guidelines
ending consultation quickly with AP
prescribing more when patient returns
seeing patients face-to-face for AP
separating patient's suffering from AP decisions
symptomology

Name
thinking of risks of AB when prescribing
unusual guideline recommendations
weighing rationales for AP and AMR
factors influencing antibiotic prescribing decisions
indications often prescribed for
investigating clinical signs
non-clinical factors
access to care
having too little time for consultations
timing of consultation influencing GP decision
patient groups at high AB-related risks
patient groups at high infection-related risks
patients appearance as factor in decisions
prescribing more to children
resources used in decision-making
consulting with colleagues
deciding based on Point-of-care tests
guidelines used
taking history and comorbidities into account
GP culture in practice
change in practice leading to higher AP
GP groups following the same pattern
having protocols for AP for all practitioners

Name
influential GPs within the practice
practice culture of consulting with colleagues
GP factors influencing decision-making
being proactive
change in personal clinical practice
changes to how GPs are trained
GPs with prescribing as a habit
personal clinical practice
professional confidence
professional discrepancies
research experience
staying up to date with guidance
variability in GP styles
younger GPs following guidance and tools more
needs for tools around improving prescribing
requirements of useful electronic tool
shortcomings of electronic tools
lack of confidence in accuracy of tool
limitations of tool to provide individualized care
showing information to back up decision-making
use of existing electronic tools
usefulness of electronic tool
using tools to justify decisions made on other basis

Name
other
future plans
knowledge of practice prescribing status
local environmental health factors
North South differences
patient population in practice
reasons provided to explain study findings for AB prescribing
GP providing explanations of study and recommendations
Questioning study quant findings
research engagement
seeing effects of public health messages
systematic change
work experience as GP
outcomes of consultations relating to AB
being confronted with adverse outcome
coping with short consultation time
effectiveness of negotiating with patients
fitting decision to guidance after-the-fact
following guidelines leading to more AP
patient getting AB from other practitioner
rethinking AP decisions after the fact
sticking with own decision
patient behaviours

Name
feeling that patients expect results
GP behaviours creating patient expectations
medicalization among patient groups
older patients accepting GP authority
patient awareness of AMR
patient expecting AB
patient groups demanding AB most
patients choosing GP based on AP behaviours
self-managing patients expecting AB less

Abbreviations:

AB	Antibiotics
AMR	Antimicrobial Resistance
AP	Antibiotic Prescribing
GP	General Practitioner
